# Phenotypic and Genetic Heterogeneity in a Thai Glucokinase MODY Family Reveals the Complexity of Young-Onset Diabetes

**DOI:** 10.3389/fendo.2021.690343

**Published:** 2021-09-01

**Authors:** Yotsapon Thewjitcharoen, Ekgaluck Wanothayaroj, Sirinate Krittiyawong, Soontaree Nakasatien, Tsz Fung Tsoi, Cadmon K. P. Lim, Juliana C. N. Chan, Thep Himathongkam

**Affiliations:** ^1^ Diabetes and Thyroid Center, Theptarin Hospital, Bangkok, Thailand; ^2^ Department of Medicine and Therapeutics, Hong Kong Institute of Diabetes and Obesity and Li Ka Shing Institute of Health Sciences, The Chinese University of Hong Kong, Prince of Wales Hospital, Shatin, Hong Kong, SAR China; ^3^ Asia Diabetes Foundation, Shatin, Hong Kong, SAR China

**Keywords:** GCK-MODY, WFS1, Apo-A1, SLC29A3, deafness, heterogeneity

## Abstract

Glucokinase-Maturity-Onset Diabetes of the Young (*GCK*-MODY) is characterized by asymptomatic, non-progressive and fasting hyperglycemia, albeit not without phenotypic variability. We used next generation sequencing (NGS) to screen for 34 MODY genes in a non-obese person with familial young-onset diabetes followed by screening in 24 family members within three generations with varying presentations of young-onset diabetes and sensorineural hearing loss. The index patient was found to carry a paternally-inherited heterozygous missense variant (c.716 A>G) of *GCK* in exon 7 with amino acid change (Q239R). This variant was associated with phenotypic heterogeneity ranging from normal glucose tolerance to diabetes with complications amongst the siblings which might be modified by obesity and chronic hepatitis B infection. Two paternally-inherited variants of *SLC29A3* encoding a nucleoside transporter protein and *Apo-A1* genes also co-segregated with glucose and lipid traits. Co-occurrence of diabetes and deafness in maternal aunts led to discovery of *WFS1* (Wolfram syndrome type 1) as a cause of non-syndromic deafness in multiple members of the maternal pedigree. Our findings highlight the complex causes of familial young-onset diabetes and the need of a multidisciplinary approach to interpret the clinical relevance of discoveries made by NGS in this era of genomic medicine.

## Introduction

Earlier studies, conducted mainly in Caucasians, suggested that Glucokinase-Maturity-Onset Diabetes of the Young (*GCK*-MODY), is a mild condition with stable fasting hyperglycemia not requiring medical treatment (1). However, there are sporadic reports on severe forms of *GCK*-MODY due to co-existence of autoimmune diabetes or obesity-driven insulin resistance (2, 3). Diagnosis of MODY is an exemplary example of precision medicine where delayed diagnosis and intervention could lead to serious complications while family screening can enable early diagnosis and treatment. Besides, patients with different MODY subtypes might respond differently to oral drugs or insulin, making precise diagnosis important in guiding treatment selection (4).

Both clinical acumen and modern technology are needed in our pursuit of precision medicine. The use of next generation sequencing (NGS) has reduced the cost and time of genetic discovery with new forms of monogenic diabetes being reported. Moreover, co- occurrence of various subtypes of MODY and mitochondrial diabetes within the same family had been reported (5, 6). Herein, we report the phenotypic and genetic heterogeneity of a large multigenerational Thai family with *GCK*-MODY.

## Materials and Methods

### Ethics Statement

This study was approved by the Ethics Committee of Theptarin Hospital. All participants gave written informed consent.

### Study Population

A total of 25 family members within three generations of young-onset diabetes with or without sensorineural hearing loss was studied. Genomic DNA was extracted from peripheral blood for sequencing. Clinical information and biochemical characteristics were documented and 75-gram oral glucose tolerance test (OGTT) was performed to verify glycemic status. Assessment of insulin resistance by homeostasis model assessment (HOMA-IR) was evaluated (7). Whole Exome Sequencing (WES) based on NGS using Human Core Exome Kit and Human RefSeq Panel from Twist Bioscience was used to screen for all coding regions including intron-exon junctions of 34 MODY genes (**Supplementary Material Table 1**) and a mitochondrial mutation for maternally-inherited deafness and diabetes (MIDD, *mt A3243G*) (GemVCare, Shatin, Hong Kong). Sanger sequencing covering all coding regions and intron-exon junctions of candidate gene was used to confirm the mutations in other family members.

### *In Silico* Analyses of Candidate Variants

Sequence data were aligned to the reference genome GRCh38 to identify variants. We evaluated possible functional significance of the variants using computational predictive programs PolyPhen-2 (http://genetics.bwh.harvard.edu/pph2) and SIFT (http://sift.jcvi.org/). Sequence variations were described using nomenclature of the Human Gene Mutation Database (HGMD). Based on the American College of Medical Genetics and Genomics (ACMG) standards and guidelines, these sequence variants were interpreted as pathogenic, likely pathogenic, variant of uncertain significance (VUS), likely benign, and benign (8).

## Results

**Figure 1:**
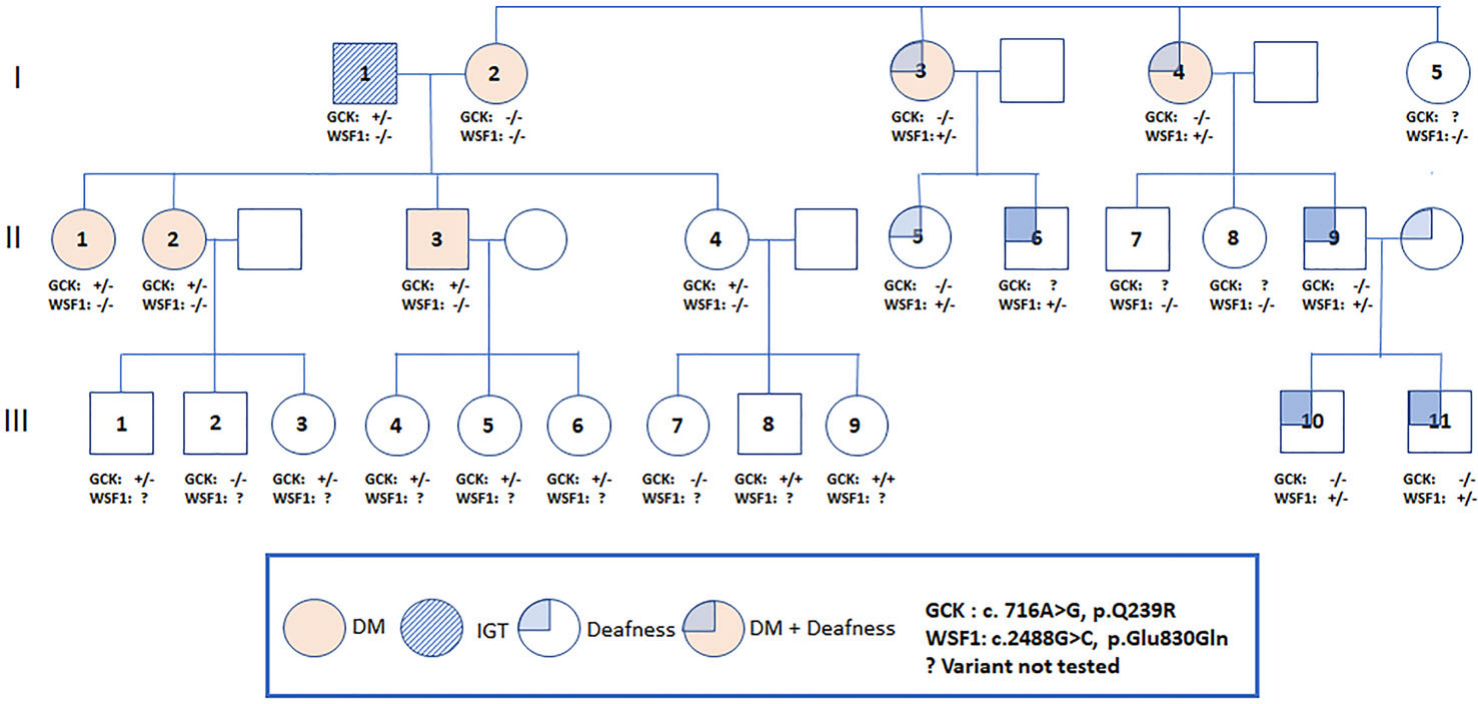
Pedigree of a Thai family with the Q239R mutation in the *GCK* gene. An arrow indicates the proband. Filled symbols indicate diabetes and non-filled symbols indicate normal or unknown glucose tolerance. Carrier status is indicated in those who underwent genotyping (+/+ = homozygous, +/- =heterozygous, -/- = non-carrier).

The proband [female, II-1, aged 41, body mass index (BMI) 23.9 kg/m^2^] was detected to have high fasting plasma glucose (FPG) of 120 mg/dL at the age of 34. She was diagnosed to have type 2 diabetes (T2D) when aged 37 with a FPG of 150 mg/dL and glycated hemoglobin (A1C) of 7.0%. Since then, she was put on metformin 1000 mg daily with a A1C range of 5.9-7.1%. Prior to the date of 75-gram OGTT, she had discontinued metformin for nearly 1 year. Her mother (I-2, aged 66) had T2D since the age of 38 with A1C of 7.5-8.7% and complicated by retinopathy and kidney disease. Her two maternal aunties (I-3, I-4) had diabetes and deafness, the latter affecting some of their family members (**Figure 1**).

The proband’s younger sister (II-2, aged 40) had insulin-treated gestational diabetes when aged 31 and was diagnosed to have T2D at the age of 38 during screening (FPG 170 mg/dL, A1C 7.7%). She had hypertension, obesity (BMI 28.6 kg/m^2^) and proteinuria and received multiple drugs with A1C of 7.5-8.3%. Autoantibody for glutamic acid decarboxylase was negative in II-1 and II-2.

We conducted a research study to investigate possible genetic causes of diabetes in this family. We used NGS to screen for all coding regions including intron-exon junctions of 34 MODY genes (**Supplementary Material Table 1**) and *mt A3243G* in I-1, I-2, II-1, II-2, II-3 and II-4 and Sanger sequencing to confirm mutations associated with diabetes and deafness in other family members.

The proband and II-2 were heterozygous for a previously reported missense variant (c.716 A>G) of *GCK* gene with amino acid change (Q239R) (9). The allele frequency was 0.00054 (The Genome Aggregation Database, *gnomAD*, East Asians) (**Supplementary Material Table 2**) and located within exon 7, a hotspot for mutations. This variant was classified as VUS by the ACMG guidelines due to its high frequency in Asian population (**Supplementary Material Table 2**). Both patients were also heterozygous for a missense variant (c.640 G>A) of *SLC29A3* in exon 5 with amino acid change (V214M). *In-silico* analysis predicted damaging effect, classified as VUS by the ACMG guidelines. The allele frequency was less than 0.00962 (*gnomAD*, East Asians) and not found in 50 Thai healthy subjects.

We offered 75-gram OGTT to the father (I-1) in his 70s and 2 siblings (II-3, II-4) in their 40s with no known history of diabetes. The father (I-1) had impaired glucose tolerance (IGT), the younger brother (II-3) had diabetes and the youngest sister (II-4) had normal glucose tolerance. The results of OGTT and HOMA-IR were summarized in **Table 1**. The father and all children were heterozygous for *GCK* Q239R and *SLC29A3* V214M variants. The mother (I-2) carried neither of these two variants (**Figure 1**). Due to the heterogeneous phenotypes amongst the carriers of these two VUS, we screened for surface antigen of hepatitis B (HBsAg) which had been reported to increase the risk of T2D by 3-fold in Asian carriers versus non-carriers (10). The mother I-2 (BMI 23.8 kg/m^2^) and sibling II-3 (BMI, 31.0 kg/m^2^) and II-4 (BMI, 26.0 kg/m^2^) were HBsAg carriers. Sibling II-3 with both obesity and positive HBsAg status had diabetes. In the third generation, amongst the 9 children (age 2 to 12), 5 were heterozygous and 2 were homozygous carriers for *GCK* Q239R. None of them had history of diabetes although formal 75g OGTT was not performed.

**Table 1:** Detailed clinical and laboratory data in family members with GCK-MODY.

	I-1	II-1*	II-2	II-3	II-4
	Father	Index case	Sibling	Sibling	Sibling
Gender	M	F	F	M	F
Present age	72	43	41	40	37
Dysglycemia status	IGT	IGT	DM	DM	No
Age of first diagnosis of dysglycemia	72	34	31 (GDM)	40	–
Body mass index (kg/m^2^)	31.0	23.9	28.6	31.0	26.0
HBV carrier	No	No	No	Yes	Yes
75 g OGTT, 0 min (mg/dL)	107	102	208	104	90
75 g OGTT, 120 min (mg/dL)	175	175	324	204	122
Plasma insulin (µU/mL)	22.3	5.9	75.2	24.0	14.5
HOMA-IR	5.8	1.5	38.6	6.2	3.2
HbA1C (% NGSP)	6.1%	5.1%	8.0%	5.6%	4.7%
eGFR (mL/min/1.73 m^2^)	104	107	125	116	98
Triglyceride (mg/dL)	220	155	232	154	138
HDL-cholesterol (mg/dL)	34	48	30	28	34
LDL-cholesterol (mg/dL)	116	150	133	187	127
Treatment for diabetes	None	None	OGLDs	None	None
Statin treatment	Yes	Yes	Yes	Yes	No

*Index case.

All results refer to the latest available results.

GDM, Gestational Diabetes Mellitus; eGFR, estimated Glomerular Filtration Rate; HBV, Hepatitis B Virus; HOMA-IR, Homeostatis Model Assessment - Insulin Resistance; IFG, Impaired Fasting Glucose; IGT, Impaired Glucose Tolerance; NGSP, National Glycohemoglobin Standardization Program; OGLDs, Oral Glucose Lowering Drugs; OGTT, Oral Glucose Tolerance test.

During screening for cardiometabolic risk factors, we noted isolated low HDL-C levels (28-34 mg/dl) in the proband’s father and 3 siblings (II-2,3,4). Since Apo A-1 is the main lipoprotein of HDL-C (11), we performed additional NGS of the *Apo-A1* gene and detected co-segregation of the paternally-inherited mutation of p.Gln156AlafsTer11 in all siblings with low HDL-C levels. This novel mutation created a premature stop codon which can lead to nonsense mediated decay of the mRNA and is likely to be pathogenic by ACMG guidelines.

The two maternal aunts (I-3, I-4) with diabetes and deafness did not carry the *GCK* or *SLC29A3* mutations or m*tA3243G* mutation. The mother of proband (I-2) had diabetes without deafness and was negative for all 3 mutations. Using the MODY panel, both maternal aunts were found to carry a mutation in the *WFS1* (Wolfram syndrome type 1) known to be associated with diabetes and deafness (12). Both maternal aunts but not the proband’s mother were heterozygous for an amino-acid changing mutation (p.E830Q). This novel variant was predicted to damage protein functions and classified as pathogenic by the ACMG guidelines. No additional mutations of the 34 MODY genes were detected in the mother or maternal aunts. Using Sanger sequencing, the *WFS1* mutation (p.E830Q) was detected in 5 of the 8 family members of the 2 maternal aunts which co-segregated with deafness. None of these family members reported history of diabetes.

## Discussion

Age, obesity, insulin resistance and other non-genetic factors can modify clinical presentation of MODY (2, 13). Using a MODY panel and NGS, we discovered co-segregation of 2 paternally-inherited mutations of *GCK* and *SLC29A3* with diabetes and 1 paternally-inherited mutation of Apo-A1 with low HDL-C amongst the children. The presence of deafness and diabetes in the maternal aunts led to the discovery of a *WFS-1* mutation in the maternal pedigree, although none of the members had diabetes. Meanwhile, the cause of diabetes in the mother remained uncertain.

There are many lessons learnt in this Thai family. Using a MODY panel, we discovered a paternally-inherited *GCK* variant to be the cause of diabetes in a non-obese patient with familial young-onset diabetes who subsequently discontinued metformin due to mild disease. However, the phenotypic variability amongst the family members raised the possibility of other modifying factors to influence the typical non-progressive clinical course of *GCK*-MODY. Although the missense variant (c.716 A>G) of *GCK* gene with amino acid change (Q239R) detected in this family was considered to be a VUS due to its high frequency in Asian population, this mutation had been reported in other Chinese families of young-onset diabetes (9). In this Thai family, despite all carrying the same mutation, both the father and index case had IGT (after discontinuing metformin for 1 year) while 2 siblings had diabetes with varying degrees of obesity and HBV status which might contribute towards the phenotypic heterogeneity. Other reports on correlations between molecular severity of *GCK* mutations and glucose tolerance yielded mixed results (13–15). In a study of Italian children with *GCK*-MODY, individual differences in insulin sensitivity but not molecular severity of *GCK* mutations affected the 2-hour post-load blood glucose level during OGTT (13).

Using a MODY panel and NGS, we discovered additional paternally-inherited mutations implicated in syndromic diabetes and lipid metabolism with potential pathogenic significance although these findings should be interpreted with caution. Homozygous carriers of *SLC29A3* mutation were reported to have the rare H syndrome characterized by pigmented hypertrichotic dermatosis, insulin-dependent diabetes and neuroendocrine dysfunction (16). However, no report of familial diabetes had been reported in heterozygous carriers of *SLC29A3* mutation and its significance remains unclear in this *GCK*-MODY family. Given the frequent co-occurrence of low HDL-C with diabetes (17), the co-segregation of the paternally-inherited rare mutant of the *Apo-A1* gene (18) with lipid traits might be coincidental rather than causal. A recent mendelian randomization study did not show causal relationship between genetically-reduced HDL-C and increased risk of type 2 diabetes (19).

With increasing affordability of NGS, our study also revealed its utility in uncovering rare causes of maternally-inherited diabetes and deafness, traditionally considered due to mitochondrial mutations (20). Both maternal aunts with deafness and diabetes carried a novel mutation in the *WFS1* gene (21). This gene encodes for the wolframin protein implicated in calcium metabolism and could explain the autosomal dominant inheritance of hearing loss in all affected family members in the maternal pedigree. However, we were not able to detect mutations in this MODY panel which could explain the young-onset diabetes with complications in the mother of the index case.

## Conclusions

In this Thai family, we discovered a paternally-inherited *GCK* mutation with phenotypic heterogeneity possibly modified by other non-genetic factors including timing of diagnosis and treatment. The use of NGS has also led to the discovery of paternally-inherited mutations co-segregated with glucose and lipid traits and a novel mutation of *WFS1* gene in the maternal pedigree with diabetes and deafness. Thus, apart from gene-environment-lifestyle-treatment interactions that can modify clinical presentation, the discovery of these rare variants through NGS requires careful interpretation by a multidisciplinary team including clinicians, geneticists, molecular biologists and data scientists in our advice of patients with or at risk of having monogenic diabetes often with partial penetrance and complex inheritance with regards to diagnosis, treatment and genetic counselling.

## Data Availability Statement

The raw data supporting the conclusions of this article will be made available by the authors, without undue reservation.

## Ethics Statement

The studies involving human participants were reviewed and approved by the Ethics Committee of Theptarin Hospital. Written informed consent to participate in this study was provided by the participants’ legal guardian/next of kin.

## Author Contributions

YT and JC wrote the manuscript. TT and CL conceived and performed genetic analysis. YT, EW, SN, and TH were involved in the care of patients. SK, CL, JC, and TH reviewed the manuscript. YT and JC are the co-guarantors of this work and, as such, have full access to all the data in the study and take responsibility for the integrity of the data and the accuracy of the data analysis. All authors contributed to the article and approved the submitted version.

## Funding

This work was supported by a grant for promoting intramural research in Theptarin Hospital (Grant No. 1/2563).

## Conflict of Interest

CL is the Chief Scientific Officer at GemVCare, a diabetes genetic testing laboratory, which was established through support from the Technology Start-up Support Scheme for Universities (TSSSU) funded by the Hong Kong Government Innovation and Technology Commission (ITC). JC and CL are co-founders of GemVCare.

The remaining authors declare that the research was conducted in the absence of any commercial or financial relationships that could be construed as a potential conflict of interest.

## Publisher’s Note

All claims expressed in this article are solely those of the authors and do not necessarily represent those of their affiliated organizations, or those of the publisher, the editors and the reviewers. Any product that may be evaluated in this article, or claim that may be made by its manufacturer, is not guaranteed or endorsed by the publisher.
