# Supplementary material for: Phenotypic and Genetic Heterogeneity in a Thai Glucokinase MODY Family Reveals the Complexity of Young-Onset Diabetes
Source: Front Endocrinol (Lausanne). 2021 Sep 1;12:690343. doi: 10.3389/fendo.2021.690343 (PMC8498575; doi:10.3389/fendo.2021.690343)
Supplement: Supplementary file 1 [file DataSheet_1.docx]

**Supplementary material**

**Table 1**. The panel consists of known genes for monogenic diabetes including all exomes and intron-exon junction detected by next generation sequencing. The selection of genes was based on Ellard S, et al. (Diabetologia 2013;56:1958-6) and previously published results.

| *ABCC8* | ATP-binding cassette transporter sub-family C member 8 |
| --- | --- |
| *AKT2* | AKT Serine/Threonine Kinase 2 |
| *APPL1* | Adaptor Protein, Phosphotyrosine Interacting With PH Domain And Leucine Zipper 1 |
| *CEL* | Carboxyl ester lipase |
| *CISD2* | CDGSH Iron Sulfur Domain 2 |
| *DCAF17* | DDB1 And CUL4 Associated Factor 17 |
| *DNAJC3* | DnaJ Heat Shock Protein Family (Hsp40) Member C3 |
| *DYRK1B* | Dual Specificity Tyrosine Phosphorylation Regulated Kinase 1B |
| *GATA4* | GATA Binding Protein 4 |
| *GATA6* | GATA Binding Protein 6 |
| *GCK* | Glucokinase |
| *HNF1A* | Hepatocyte nuclear factor-1 alpha |
| *HNF1B* | Hepatocyte nuclear factor-1 beta |
| *HNF4A* | Hepatocyte nuclear factor-4 alpha |
| *INS* | Insulin |
| *INSR* | Insulin receptor |
| *KCNJ11* | Potassium Inwardly Rectifying Channel Subfamily J Member 11 |
| *LMNA* | Lamin A/C |
| *NEUROD1* | Neurogenic differentiation 1 |
| *PAX4* | Paired box gene 4 |
| *PAX6* | Paired box gene 6 |
| *PCBD1* | Pterin-4 Alpha-Carbinolamine Dehydratase 1 |
| *PDX1* | Pancreatic And Duodenal Homeobox 1 |
| *PIK3R1* | Phosphoinositide-3-Kinase Regulatory Subunit 1 |
| *PLIN1* | Perilipin 1 |
| *POLD1* | DNA Polymerase Delta 1 |
| *PPARG* | Peroxisome proliferator-activated receptor |
| *PPP1R15B* | Protein Phosphatase 1 Regulatory Subunit 15B |
| *RFX6* | Regulatory factor X6 |
| *SLC29A3* | Solute Carrier Family 29 Member 3 |
| *TRMT10A* | TRNA Methyltransferase 10A |
| *WFS1* | Wolframin ER Transmembrane Glycoprotein |
| *ZBTB20* | Zinc Finger And BTB Domain Containing 20 |
| *ZFP57* | Zinc finger protein 57 homolog |

**Table 2.** Summary of allele frequency of Q239R *GCK* variant and V214M *SLC29A3* variant in each population according to The Genome Aggregation Database (gnomAD)

| **Population** | ***GCK***  **Q239R** | ***SLC29A3***  **V214M** |
| --- | --- | --- |
| East Asian | 0.0005441 | 0.009622 |
| South Asian | 0.000 | 0.00006533 |
| African/ African-American | 0.000 | 0.00004004 |
| European (non-Finnish) | 0.000 | 0.00001548 |
| Latino/ Admixed American | 0.000 | 0.000 |
| Ashkenazi Jewish | 0.000 | 0.000 |
| European (Finnish) | 0.000 | 0.000 |
| Other | 0.000 | 0.000 |
